# External Validation of a Measurement Tool to Assess Systematic Reviews (AMSTAR)

**DOI:** 10.1371/journal.pone.0001350

**Published:** 2007-12-26

**Authors:** Beverley J. Shea, Lex M. Bouter, Joan Peterson, Maarten Boers, Neil Andersson, Zulma Ortiz, Tim Ramsay, Annie Bai, Vijay K. Shukla, Jeremy M. Grimshaw

**Affiliations:** 1 Community Information and Epidemiological Technologies (CIET), Ottawa, Ontario, Canada; 2 Institute for Research in Extramural Medicine (EMGO Institute), Vrije Universiteit (VU) University Medical Center, Amsterdam, The Netherlands; 3 Executive Board, Vrije Universiteit (VU) University Amsterdam, Amsterdam, The Netherlands; 4 Clinical Epidemiology Program, Ottawa Health Research Institute, University of Ottawa, Ontario, Canada; 5 Department of Clinical Epidemiology and Biostatistics, Vrije Universiteit (VU) University Medical Center, Amsterdam, The Netherlands; 6 Centro de Investigación de Enfermedades Tropicales (CIET), Universidad Autónoma de Guerrero, Acapulco, Mexico; 7 Epidemiological Research Institute, National Academy of Medicine, Buenos Aires, Argentina; 8 Canadian Agency for Drugs and Technologies in Health (CADTH), Ottawa, Ontario, Canada; University of Toronto, Canada

## Abstract

**Background:**

Thousands of systematic reviews have been conducted in all areas of health care. However, the methodological quality of these reviews is variable and should routinely be appraised. AMSTAR is a measurement tool to assess systematic reviews.

**Methodology:**

AMSTAR was used to appraise 42 reviews focusing on therapies to treat gastro-esophageal reflux disease, peptic ulcer disease, and other acid-related diseases. Two assessors applied the AMSTAR to each review. Two other assessors, plus a clinician and/or methodologist applied a global assessment to each review independently.

**Conclusions:**

The sample of 42 reviews covered a wide range of methodological quality. The overall scores on AMSTAR ranged from 0 to 10 (out of a maximum of 11) with a mean of 4.6 (95% CI: 3.7 to 5.6) and median 4.0 (range 2.0 to 6.0). The inter-observer agreement of the individual items ranged from moderate to almost perfect agreement. Nine items scored a kappa of >0.75 (95% CI: 0.55 to 0.96). The reliability of the total AMSTAR score was excellent: kappa 0.84 (95% CI: 0.67 to 1.00) and Pearson's R 0.96 (95% CI: 0.92 to 0.98). The overall scores for the global assessment ranged from 2 to 7 (out of a maximum score of 7) with a mean of 4.43 (95% CI: 3.6 to 5.3) and median 4.0 (range 2.25 to 5.75). The agreement was lower with a kappa of 0.63 (95% CI: 0.40 to 0.88). Construct validity was shown by AMSTAR convergence with the results of the global assessment: Pearson's R 0.72 (95% CI: 0.53 to 0.84). For the AMSTAR total score, the limits of agreement were −0.19±1.38. This translates to a minimum detectable difference between reviews of 0.64 ‘AMSTAR points’. Further validation of AMSTAR is needed to assess its validity, reliability and perceived utility by appraisers and end users of reviews across a broader range of systematic reviews.

## Introduction

High quality systematic reviews are increasingly recognized as providing the best evidence to inform health care practice and policy [1]. The quality of a review, and so its worth, depends on the extent to which, scientific review methods were used to minimize the risk of error and bias. The quality of published reviews can vary considerably, even when they try to answer the same question [2]. As a result, it is necessary to appraise their quality (as is done for any research study) before the results are implemented into clinical or public health practice. Much has been written on how best to appraise systematic reviews, and while there is some variation on how this is achieved, most agree on key components of the critical appraisal [3]. Methodological quality can be defined as the extent to which the design of a systematic review will generate unbiased results [4].

Several instruments exist to assess the methodological quality of systematic reviews [5], but not all of them have been developed systematically or empirically validated and have achieved general acceptance. The authors of this paper acknowledge that the methodological quality and reporting quality for systematic reviews is very different. The first, *methodological quality*, considers how well the systematic review was conducted (literature searching, pooling of data, etc.). The second, *reporting quality*, considers how well systematic reviewers have reported their methodology and findings. Existing instruments often try to include both types of methods without being conceptually clear about the differences.

In an attempt to achieve some consistency in the evaluation of systematic reviews we have developed a tool to assess their methodological quality. This builds on previous work [6], and is based on empirical evidence and expert consensus. A measurement tool to assess systematic reviews (AMSTAR) was highly rated in a recent review (personal communication) of quality assessment instruments performed by the Canadian Agency for Drugs and Technologies in Health (CADTH). In this study we present the results of an external validation of AMSTAR using data from a series of systematic reviews obtained from the gastroenterology literature.

## Methods

The characteristics and basic properties of the instrument have been described elsewhere [7]. Briefly, a 37-item initial assessment tool was formed by combining a) the enhanced Overview Quality Assessment Questionnaire (OQAQ) scale, b) a checklist created by Sacks, and c) three additional items recently judged by experts in the field to be of methodological importance. In its development phase the instrument was applied to 99 paper-based and 52 electronic systematic reviews [6] [7]. Exploratory factor analysis was used to identify underlying components. The results were considered by methodological experts using a nominal group process to reduce the number of items and design an assessment tool with face and content validity. This process lead to an 11-item instrument [7]. A description of the instrument is provided in Annex S1.

### External validity

For our validation test set we chose to use systematic reviews or meta-analyses in the area of gastroenterology, specifically upper gastrointestinal. CADTH's informational specialist searched electronic bibliographic databases (i.e. Medline, Central and EMBASE) up to and including 2005. A total of 42 systematic reviews met the *a priori* criteria and were included [8]. This sample included seven electronic Cochrane systematic reviews and 35 paper-based non-Cochrane reviews. The topics of the reviews ranged across the spectrum of GI problems like dyspepsia, gastro-esophageal reflux disease (GERD), peptic ulcer disease (PUD), and also GI drug interventions such as H2 receptor antagonists and proton pump inhibitors [9]–[50].

Two CADTH assessors from two review groups (SS and FA, AL and CY) independently applied AMSTAR to each review and reached agreement on the assessment results. To assess construct validity, two reviewers (JP, ZO) plus a clinician and/or methodologist (MB, DF, DP, MO, and DH) applied a global assessment to each review [51] (Annex S2).

### Agreement and reliability

**Figure 1:**
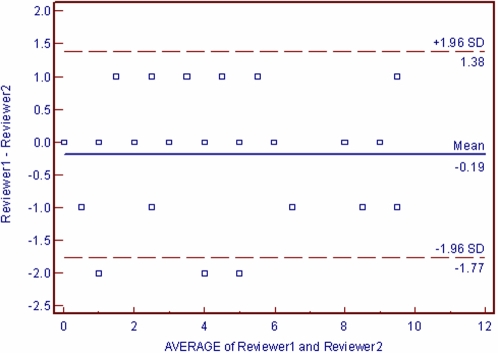
Bland and Altman limits of agreement plot for AMSTAR scores.

We calculated an overall agreement score using the weighted Cohen's kappa, as well as one for each item [52] (Table 1). Bland and Altman's limits of agreement methods were used to display agreement graphically [53], [54] (Fig. 1). We calculated the percentage of the theoretical maximum score. Pearson's Rank correlation coefficients were used to assess reliability of this total score. For comparisons of rating the methodological quality we calculated chance-corrected agreement (using kappa) and chance-independent agreement (using Φ) [52], [55], [56]. We accepted a correlation of >0.66. We further scrutinized items and reviews with kappa scores below 0.66 [52]. Kappa values of less than 0 rate as less than chance agreement; 0.01–0.20 slight agreement; 0.21–0.40 fair agreement; 0.41–0.60 moderate agreement; 0.61–0.80 substantial agreement; and 0.81–0.99 almost perfect agreement [52], [57]. We calculated PHI Φ for each question [55], [58].

**Table 1:** Assessment of the inter-rater agreement for AMSTAR

Items	Kappa (95% CI)	PHI Φ
1. Was an ‘a priori’ design provided?	0.75 (0.55 to 0.96)	0.76
2. Was there duplicate study selection and data extraction?	0.81 (0.63 to 0.99)	0.83
3. Was a comprehensive literature search performed?	0.88 (0.73 to 1.00)	0.89
4. Was the status of publication (i.e. grey literature) used as an inclusion criterion?	0.64 (0.40 to 0.88)	0.64
5. Was a list of studies (included and excluded) provided?	0.84 (0.67 to 1.00)	0.84
6. Were the characteristics of the included studies provided?	0.76 (0.55 to 0.96)	0.76
7. Was the scientific quality of the included studies assessed and documented?	0.90 (0.77 to 1.00)	0.91
8. Was the scientific quality of the included studies used appropriately in formulating conclusions?	0.51 (0.25 to 0.78)	0.56
9. Were the methods used to combine the findings of studies appropriate?	0.80 (0.63 to 0.99)	0.80
10. Was the likelihood of publication bias assessed?	0.85 (0.64 to 1.00)	0.85
11. Were potential conflicts of interest included?	1.00 (100% no)	1.00
**Overall Score**	0.84 (0.67 to 1.00)	0.85

### Construct validity

We assessed construct validity (i.e. evaluation of a hypothesis about the expected performance of an instrument) by converting the total mean score (mean of the two assessors) for each of the 42 reviews to a percentage of the maximum score for AMSTAR and of the maximum score of the global assessment instrument. We used Pearson's Rank correlation coefficients, Pearson's R and Kruskal-Wallis test to further explore the impact of the following items on the construct validity of AMSTAR: a) Cochrane systematic review vs. non-Cochrane systematic reviews [59], [60], b) journal type [61], c) year of publication [62], d) conflict of interest [63], e) impact factor [64], and number of pages [64]. We studied these in the context of a priori hypotheses concerning the correlation of AMSTAR scores. Because of the nature of their development, we anticipated that Cochrane systematic reviews would have higher quality scores than non-Cochrane systematic reviews and those electronic or general journals would score higher than specialist journals. We reported on impact factors for these journals. We hypothesized that reviews published more recently would be of higher quality than those published earlier. In addition, we anticipated that reviews declaring a conflict of interest might have lower quality scores [63], [64].

We assessed the practicability of the new instrument by recording the time it took to complete scoring and the instances where scoring was difficult. We interviewed assessors (N = 6) to obtain data on clarity, ambiguity, completeness and user-friendliness.

We used SPSS (versions 13 and 15) and MedCalc for Windows, version 8.1.0.0.

## Results

The 42 reviews included in the study had a wide range of quality scores. The overall scores estimated by the AMSTAR instrument ranged from 0 to 10 (out of a maximum of 11) with a mean of 4.6 (95% CI: 3.7 to 5.6; median 4.0 (range 2.0 to 6.0). The overall scores for the global assessment instrument ranged from 2 to 7 (out of a maximum score of seven) with a mean of 4.43 (95% CI: 3.6 to 5.3) and median 4.0 (range 2.5 to 5.3).

### Agreement and Reliability

The reliability of the total AMSTAR score between two assessors (the sum of all items answered ‘yes’ scored as 1, all others as 0) was (kappa 0.84 (95% CI: 0.67 to 1.00, Φ = 0.85) and Pearson's R 0.96 (95% CI: 0.92 to 0.98). The inter-rater agreement (kappa) between two raters, for the global assessment was 0.63 (95% CI: 0.40 to 0.88).

Items in AMSTAR displayed levels of agreement that ranged from moderate to almost perfect; nine items scored a kappa of >0.75 (0.55 to 0.96 (and Φ >0.76). Item 4 had a kappa of 0.64 (0.40 to 0.88) Φ = 0.64 and item 8 a kappa of 0.51(0.25 to 0.78 Φ = 0.56). The reliability of the total AMSTAR score was excellent (kappa 0.84 (95% CI: 0.67 to 1.00 and Pearson's R 0.96 (95% CI: 0.92 to 0.98). For the AMSTAR total score, the limits of agreement were −0.19±1.38 (Fig. 1).

The mean age of our reviewers was 40.57, median 43. Fifty-seven percent were identified as experts in methodology and 43% were identified as content experts in the field.

### Construct validity

Expressed as a percentage of the maximum score, the results of AMSTAR converged with the results of the global assessment instrument [Pearson's Rank Correlation Coefficient 0.72 (95% CI: 0.53 to 0.84)]. AMSTAR scoring also upheld our other *a priori* hypotheses. The sub-analysis revealed that Cochrane reviews had significantly higher scores than paper-based reviews with a (R = 37.21 n = 7) for Cochrane reviews and (R = 18.36 n = 35) for paper-based (P<0.0002). Cochrane reviews (R = 37.21 n = 7) also scored higher than reviews published in general journals (R = 25.77 n = 11) and specialty journals (R = 14.96, n = 24) (P<0.0001). Reviews published from 2000 onward had higher AMSTAR scores than earlier reviews (R = 25.20, n = 25 vs. R = 13.12, n = 17; P = 0.0002).

The journals had the following overall summary statistics for the impact factors: mean 5.88 (95% CI: 3.9 to 7.9) median 3.3 (lowest value 1.4, highest value 23.9). There is no statistical association between AMSTAR score and impact factor (Pearson's R (0.555 P = 0.7922)). There was however a significant association found with the number of pages and AMSTAR scores (Pearson's R (0.5623 P = 0.0001 n = 42). We found no association (R 0.1773 P = 0.0308) when we removed the outliers (i.e. systematic reviews with higher page numbers).

Conflict of interest was poorly presented. Of the 42 reviews assessed, no study had appropriately declared their conflict of interest. Therefore, we were unable to assess whether or not funding had a positive or negative effect on the AMSTAR score.

### Practicability

Both AMSTAR and the global assessment required on average 15 minutes to complete, but with the latter, assessors expressed difficulty in reaching a final decision in the absence of comprehensive guidelines. In contrast, AMSTAR was well received.

## Discussion

### Principal findings

This paper describes an external validation of AMSTAR. This new measurement tool to assess methodological quality of systematic reviews showed satisfactory inter-observer agreement, reliability and construct validity in this study. Items in AMSTAR displayed levels of agreement that ranged from moderate to almost perfect. The reliability of the total AMSTAR score was excellent. Construct validity was shown by AMSTAR convergence with the results of the global assessment instrument.

We found a significant association between number of published pages and overall AMSTAR score, suggesting that the longer the manuscript, the higher the quality score. It should be interpreted with caution given the fact that only a couple of the longer reviews largely drive the hypothesis tests. We found no association when the outliers were removed from the dataset. We did not find an association between AMSTAR score and impact factor.

The AMSTAR instrument was developed pragmatically using previously published tools and expert consensus. The original 37 items were reduced to an 11- item instrument addressing key domains; the resulting instrument was judged by the expert panel to have face and content validity [7].

### Strengths and weaknesses of the study

This is a prospective external validation study. We compared the new instrument to an independent and reliable gold standard designed for assessing the quality of systematic reviews, allowing multiple testing of convergent validity.

The analytical methods for assessing quality and measuring agreement amongst assessors need further discussion and development. We calculated chance-corrected agreement, using the kappa statistic [57], [65]. While avoiding high levels of agreement due to chance, kappa has its own limitations that have lead to academic criticism [66], [67]. One of the major difficulties with kappa is that when the proportion of positive ratings is extreme, the possible agreement above chance agreement is small and it is difficult to achieve even moderate values of kappa. Thus, if one uses the same raters in a variety of settings, as the proportion of positive ratings becomes extreme, kappa will decrease even if the manner in which the assessors rate the quality does not change. To address this limitation, we also calculated chance-independent agreement using PHIΦ, a relatively new approach to assessing observer agreement [55], [58].

We were unable to test our convergent validity hypothesis about conflict of interest because of missing data in the systematic reviews and primary studies. This highlights the need for journals and journal editors to require that the information is provided.

Our results are based on a small sample of systematic reviews in a particular clinical area and a relatively small number of AMSTAR assessors. There is a need for replication in larger and different data sets with more diverse appraisers.

### Possible mechanisms and implications for clinicians or policymakers

Existing systematic review appraisal instrument did not reflect current evidence on potential sources of bias in systematic reviews and were generally not validated. The best available instrument prior to the development of AMSTAR was OQAQ which was formally validated. However, users of OQAQ frequently had to develop their own rules for operationalizing the instrument and OQAQ does not reflect current evidence on sources of potential bias in systematic reviews (for example funding source and conflict of interest [68], [69], [70]).

Quality assessment instruments can focus on either *reporting quality* (how well systematic reviewers have reported their methodology and findings (internal validity) or *methodological quality* (how well the systematic review was conducted (literature searching, pooling of data, etc.). It is possible for a systematic review with poor methodological quality to have good reporting quality. For this reason, the AMSTAR items focus on methodological quality.

Decision-makers have spent the last ten years trying to work out the best way to use the enormous amounts of systematic reviews available to them. They can hardly know where to start when deciding whether the relevant literature is valid and of the highest quality. AMSTAR is a user friendly methodological quality assessment that has the potential to standardize appraisal of systematic reviews. Early experience suggests that relevant groups are finding the instrument useful.

### Unanswered questions and future research

Further validation of AMSTAR is needed to assess its validity, reliability and perceived utility by appraisers and end users of reviews across a broader range of systematic reviews. We need to assess the responsiveness of AMSTAR looking at its sensitivity to discriminate between high and low methodological quality reviews.

We need to assess the applicability of AMSTAR for reviews of observational (diagnostic, etiological and prognostic) studies and if necessary develop AMSTAR extensions for these reviews.

We plan to update AMSTAR as new evidence regarding sources of bias within systematic reviews becomes available.

## Supporting Information

Annex S1 AMSTAR is a measurement tool created to assess the methodological quality of systematic reviews.
(0.04 MB DOC)

Annex S2 Global assessment rating
(0.03 MB DOC)
