# Supplementary material for: External Validation of a Measurement Tool to Assess Systematic Reviews (AMSTAR)
Source: PLoS One. 2007 Dec 26;2(12):e1350. doi: 10.1371/journal.pone.0001350 (PMC2131785; doi:10.1371/journal.pone.0001350)
Supplement: Annex S2 — Global assessment rating (0.03 MB DOC) [file pone.0001350.s002.doc]

**Annex S2: Global assessment rating**

Global Assessment rating will be assessed using the following instrument.*

How would you rate the scientific quality of the overview?

| Extensive Flaws |  | Major Flaws |  | Minor Flaws |  | Minimal Flaws |
| --- | --- | --- | --- | --- | --- | --- |
|   1 |   2 |   3 |   4 |   5 |   6 |   7 |

*Oxman AD, Guyatt GH. (1991) Validation of an index of the quality of review articles. J Clin Epidemiol 44(11): 1271-78.
